# The Effects of Acute Exercise on Cognitive Functions in Patients with Schizophrenia and Healthy Adults

**DOI:** 10.1192/j.eurpsy.2026.10632

**Published:** 2026-07-31

**Authors:** A. H. Bayraktar Özdemir, E. T. Özel Kızıl, G. Ömercioğlu, A. Demirci Şahin, Z. E. Erden, D. Onar, Z. Uçar Hasanlı, M. Baştuğ

**Affiliations:** 1 Department of Psychiatry; 2 Department of Physiology, Ankara University, Ankara; 3 Department of Psychiatry, Bakirkoy Training and Research Hospital for Psychiatry, Neurology and Neurosurgery, İstanbul; 4 Department of Psychiatry, Ankara Training and Research Hospital, Ankara, Türkiye

## Abstract

**Introduction:**

Cognitive dysfunctions are considered as core features of schizophrenia and significantly affect functional outcomes. Physical exercise has emerged as a promising non-pharmacological strategy to enhance cognitive functioning in schizophrenia patients.Aerobic exercise has consistently demonstrated positive effects on domains such as attention,memory, and executive functions (Shimada et al. Psychiatry Res 2022; 314:114656). While the benefits of regular aerobic exercise are well-established, recent meta-analyses indicate that even a single session of moderate-intensity aerobic exercise can acutely enhance cognitive performance(Chang et al. Brain Res 2012; 1453:87–101).

**Objectives:**

This study aims to investigate the effects of a single session of moderate-intensity aerobic exercise on cognitive performance in individuals with schizophrenia and healthy controls,to determine whether the effects differ between groups and to better understand the underlying mechanisms.

**Methods:**

The study included 58 participants:29 individuals with schizophrenia and 29 healthy controls.It was conducted in two sessions.All participants first performed a cardiopulmonary exercise test (CPET) on a cycle ergometer to assess aerobic capacity.Target intensity for the acute exercise session was calculated using the VO₂ reserve method.Later, participants completed a second session to evaluate the acute effects of exercise on cognitive function.Cognitive tests were administered before and after the exercise and included the Trail Making Test(TMT), Digit Span Tests(DST), Auditory Verbal Learning Test(AVLT), Verbal Fluency Test(VFT), and Stroop Test(ST). A 2×2 mixed design repeated-measures ANOVA was used to examine changes in cognitive performance as well as the group effect.

**Results:**

Acute aerobic exercise significantly improved performance on ST(p=<.001). No significant group-by-time interaction or group effect was observed.TMT Part A and B completion times were reduced post-exercise(p<.001, both), with significant main effects of group in both (p<.001, both) but no interaction effects.VFT improved significantly after exercise (p=.026); the schizophrenia group showed lower overall performance(p=.003), with no significant interaction effect. In DST forward and backward performances, only exercise had significant effects (p<.001;p<.003). AVLT learning scores showed a significant group-by-time interaction (p=.027) and group effect(p=.002), with no exercise effect. Spontaneous recall showed only a significant group effect (p=.004).

**Conclusions:**

A single session of aerobic exercise improved cognitive performance of both individuals with schizophrenia and healthy controls, particularly attention and processing speed.Although patients showed lower performance, the magnitude of improvement was comparable across groups. These findings suggest that acute aerobic exercise may offer a simple, non-pharmacological strategy to enhance cognition in schizophrenia.

**Disclosure of Interest:**

None Declared

